# Supplementary material for: CHCHD10 mutations promote loss of mitochondrial cristae junctions with impaired mitochondrial genome maintenance and inhibition of apoptosis
Source: EMBO Mol Med. 2015 Dec 14;8(1):58–72. doi: 10.15252/emmm.201505496 (PMC4718158; doi:10.15252/emmm.201505496)
Supplement: Supplementary file 1 — Appendix [file EMMM-8-58-s001.pdf]

APPENDIX _ TABLE OF CONTENTS

**SUPPLEMENTARY METHODS**	**Page 2**
**LEGENDS TO APPENDIX FIGURES**	**Page 9**
**APPENDIX TABLE S1**	**Page 14**
**APPENDIX FIGURE S1**	**Page 15**
**APPENDIX FIGURE S2**	**Page 16**
**APPENDIX FIGURE S3**	**Page 17**
**APPENDIX FIGURE S4**	**Page 18**
**APPENDIX FIGURE S5**	**Page 19**
**APPENDIX FIGURE S6**	**Page 20**
**APPENDIX FIGURE S7**	**Page 21**
**APPENDIX FIGURE S8**	**Page 22**
**APPENDIX FIGURE S9**	**Page 23**
**APPENDIX FIGURE S10**	**Page 24**
**APPENDIX REFERENCES**	**Page 25**

APPENDIX

SUPPLEMENTARY METHODS

Cell culture

Skin punches were obtained after informed consent. Primary fibroblast cultures were established using standard procedures in RPMI supplemented with 10% Fetal Bovine Serum (FBS), 45μg/ml uridine and 275μg/ml sodium pyruvate. Cultures were incubated at 37°C with 5% CO_2. For galactose conditions, medium was replaced 24h before experiments by glucose-free medium containing 5mM galactose and 5mM pyruvate (Zanna et al., 2008).

HeLa cells were maintained in DMEM supplemented with penicillin (100U/ml)/streptomycin (0.1mg/ml), 10% FBS, at 37°C in a humidified atmosphere with 5% CO_2 in air. For transient transfections, HeLa cells were transfected using Lipofectamine 2000 (Invitrogen) according to the manufacturer's instructions.

OXPHOS spectrophotometric measurements

Enzymatic spectrophotometric measurements of the OXPHOS respiratory chain complexes and citrate synthase were performed at 37°C on fibroblasts according to standard procedures (Rustin et al., 1994).

Polarographic study

Polarographic studies on fibroblasts of intact cell respiration and digitonin (0.004%)-permeabilized cells mitochondrial substrate oxidation were carried out as previously described (Rustin et al., 1994).

Confocal microscopy analysis

For mitochondrial staining, cells were incubated in a 100nM solution of Mitotracker red (Invitrogen) for 15 min, medium was replaced by fibroblasts or HeLa cells culture medium incubated 2 h at 37°C and washed in PBS. The samples were fixed with paraformaldehyde

(PFA) 4% (Electron Microscopy Sciences) for 20 min at 37°C, washed with PBS, and mounted on glass slides using Prolong Gold Antifade Reagent (Molecular Probes). For immunostaining, cells were fixed with 4% PFA, washed five times with PBS and permeabilised with 2% Triton X-100. After PBS washing, coverslips were incubated with 5% Bovin Serum Albumin (BSA) for at least 45 min at room temperature (RT) before adding rabbit anti-FLAG M2 (Cell Signaling) (1/800 antibody diluted with PBS-BSA 5%), mouse anti-DNA (Progen) (1/200 antibody diluted with PBS-BSA 5%), mouse anti-TFAM (Abnova) (1/500 antibody diluted with PBS-BSA 5%) or mouse anti-cytochrome *c* conjugated with Alexa 488 (BD Biosciences) (1/200 antibody diluted with PBS-BSA 5%). The samples were incubated at RT for 1h, PBS washed, and then incubated with fluorescent secondary antibody goat anti-mouse Alexa 488 (Life Technologies) (1/1000 antibody diluted with PBS-BSA 1%) or antibody goat anti-rabbit Alexa 647 (Life Technologies) (1/1000 antibody diluted with PBS-BSA 1%) for 1h at RT. The coverslips were washed five times with PBS, mounted on glass slides using Prolong Gold Antifade Reagent (Molecular Probes) and analyzed using a Zeiss LSM510 meta confocal laser-scanning microscope.

Mitochondrial network and nucleoid analysis

The images were deconvolved with Huygens Essential Software^{TM} (Scientific Volume Imaging) using a theoretically calculated point spread function (PSF) for each of the dyes. All selected images were iteratively deconvolved with a maximum iterations scored 40 and a quality threshold at 0.05. The deconvolved images were analysed with Huygens Essentiel Software^{TM} with the following standardised set of parameters: threshold = 25% and seed = 0% and garbage = 5 or 10 in both fibroblasts and HeLa cells for mitochondrial network analysis; threshold = 5%, seed = 5% and garbage = 5 in both fibroblasts and HeLa cells for nucleoid analysis. The quantitative data were further analysed in Microsoft Excel and GraphPad Prism 5 (GraphPad Software). Mitochondrial network length and nucleoid

parameters were quantified for three independent experiments (30-35 randomly-selected individual cells per experiment). Data are represented as mean ± S.E.M. Statistical analyses were performed by Student's unpaired t-test using GraphPad Prism 5 (GraphPad Software).

Plasmid constructions

FLAG-tagged CHCHD10 plasmids coding for wild-type (CHCHD10^{WT}) and mutant (CHCHD10^{S59L}) cDNAs were previously described (Bannwarth et al., 2014). The CHCHD10^{P34S} plasmid has been generated using the QuickChange-XL Site-Directed Mutagenesis kit (Agilent) from pCMV-3Tag-3A-CHCHD10^{WT} with following primers: 5'-GGCAGCCGCCTCAGCCCCCGC-3' (forward) and 5'-GCGGGGGCTGAGGCGGCTGCC-3' (reverse). All constructs were verified by DNA sequencing.

Transmission electron microscopy

For ultrastructural analysis, cells were fixed in 1.6% glutaraldehyde in 0.1M phosphate buffer, rinsed in 0.1M cacodylate buffer, post-fixed for 1h in 1% osmium tetroxide and 1% potassium ferrocyanide in 0.1M cacodylate buffer to enhance the staining of membranes. Cells were rinsed in distilled water, dehydrated in alcohols and lastly embedded in epoxy resin. Contrasted ultrathin sections (70 nm) were analyzed under a JEOL 1400 transmission electron microscope mounted with a Morada Olympus CCD camera.

Mitochondrial fusion assay

Mitochondrial fusion was examined using mitochondria-targeted photoactivatable GFP (mitoPAGFP), as described (Karbowski et al., 2004). The matrix-targeted presequence from Su9 (Wakabayashi et al., 2009) was fused to the N-terminus of photoactivatable GFP (Addgene) and cloned into the lentiviral vector pHR-SIN (Kim et al., 2011). Fibroblasts were infected with lentiviral particles carrying mitoPAGFP 30 min before observation and were stained with 7nM tetramethylrhodamine ethyl ester to visualize mitochondria. MitoPAGFP was photoactivated by 405-nm light (30% power, three times) in a small region (30 x 30

pixels) using a Zeiss 780 LSM confocal microscope with an environmentally controlled chamber. Images were taken at 15-min intervals for 60 min. Fluorescence intensity of mitoPAGFP was quantified using NIH Image J for at least three independent experiments. Data are represented as mean ± S.E.M. Statistical analyses were performed by Student's unpaired t-test.

Co-immunoprecipitation experiments

Fibroblasts were homogenized in solubilization buffer (50 mM Tris-HCl pH7.4, 150 mM NaCl, 0.1% Triton and protease inhibitor (Roche)) and incubated on ice for 1h. A centrifugation for 5 min at 2600 g and 4°C was performed and the cell lysate obtained was incubated with Dynabeads® M-270 Epoxy (Life technologies) for 1 h at 4°C with gentle rotation. After this pre-clearing step, lysate was incubated 1h at 4°C with gentle rotation with rabbit polyclonal anti-mitofilin antibody (Abcam) or rabbit anti-IgG (NeoMarkers, Fisher Scientific), previously coupled to Dynabeads® M-270 Epoxy using Dynabeads® Co-Immunoprecipitation Kit (Life Technologies) as described by the manufacturer. After the incubation with the cell lysate, beads were washed three times with solubilization buffer (50 mM Tris-HCl pH7.4, 150 mM NaCl, 0.1% Triton and protease inhibitor (Roche)). Then, beads were washed with LWB 1X buffer (Dynabeads® Co-Immunoprecipitation Kit (Life Technologies)) and incubated 5 min at RT with gentle rotation. Finally, bound proteins were released with Laemmli buffer. Immunoprecipitated proteins were separated on acrylamide-SDS gels and transferred to PVDF membranes (Millipore). The membrane was probed with rabbit anti-CHCHD10 (1/500, Sigma), goat anti-CHCHD3 (1/500, Abcam) and mouse anti-Mitofilin (1/500, Abcam) antibodies. Then, the appropriate secondary antibody (Dako) was used at 1/5000 and signals were detected using a chemiluminescence system (Immobilon Western HRP Chemilumiscent substrates, Millipore).

Treatment of cells with H_2O_2

Primary fibroblasts were plated in triplicate in 100mm-diameter culture sterile dishes at 60-70% confluence 20h before treatment. H_2O_2 (30%, Sigma Aldrich, St Quentin Fallavier, France) was diluted into PBS and the concentration was determined by absorbance at 260nm as described (Shull et al., 1991). Monolayer cultures were exposed to 150µM H_2O_2 for 30 min at 37°C in serum free medium. Medium culture was replaced by complete medium culture and incubated for the indicated times. Control monolayers were mocked-treated with corresponding serum free medium alone (Yakes and Van Houten, 1997). Before genomic DNA extraction, medium culture was removed and plates were rapidly frozen in liquid N_2 and stored at -80°C.

DNA damage and repair assays

Measurement of mtDNA damage was performed using quantitative PCR (qPCR) that amplifies long DNA targets. The assay was performed essentially as described previously (Hunter et al., 2010; Santos et al., 2006). DNA lesions, including H_2O_2-induced damage, block the progression of the Taq DNA polymerase resulting in a decreased amplification of a target sequence (Ballinger et al., 1999; Yakes and Van Houten, 1997). For the PCR to be quantitative, it is necessary to amplify a given amount of template at a cycle number that is within exponential range of the PCR. Therefore, for each sample, 3 separate PCR reactions were performed with 3 different cycle numbers to determine quantitative conditions. PCR conditions are available on request and the following primers were used to amplify a 15.6kb fragment of human mtDNA (sense primer 5'-CCC ACA GTT TAT GTA GCT TAC CTC CTC A-3' and reverse primer 5'-TTG ATT GCT GTA CTT GCT TGT AAG CAT G -3').

PCR quantification was normalized with a small target PCR not affected by H_2O_2 treatment. A human 172bp mtDNA fragment was amplified using a sense primer (5'-GAA TTG TGT AGG CGA ATA GG- 3') and a reverse primer (5'-CTA CAC AAT CAA AGA CGC CC-3'). All PCR reactions were quantified using Quant-iT PicoGreen dsDNA reagent (Invitrogen)

which becomes fluorescent upon binding to DNA. 5µl of each PCR reaction were diluted in TE buffer 1X (45µl) containing 1X Quant-iT PicoGreen dsDNA reagent. Standard curve was performed as described by the manufacturer. Fluorescence quantification was performed with a LightCycler LC480 apparatus. Relative PCR products of mtDNA were normalized to mtDNA copy number. The relative PCR product, which represents the relative level of oxydative DNA damage, was calculated by dividing the raw fluorescence value of a H_2O_2-treated sample by that of the corresponding H_2O_2-untreated sample.

mtDNA copy number

The mtDNA content was determined by real-time PCR using TaqMan probes as previously described (Bannwarth et al., 2012). The ratio of mtDNA copy number to nuclear DNA was used as a measure of mtDNA content in each specimen.

Western blot analysis

5-25 µg of total protein extracts were separated on acrylamide-SDS gels and transferred to PVDF membranes (Millipore). Specific proteins were detected by using mouse anti-FLAG M2 (1/1000, Cell Signaling), goat anti-Hsp60 (1/40000, Santa Cruz Biotechnology) and rabbit anti-GAPDH (1/5000, Abcam) antibodies or MitoProfile® total OXPHOS human WB antibody cocktail (1/1000, Abcam). Anti-mouse secondary antibodies (Dako) were used at 1/5000 and signals were detected using a chemiluminescence system (Immobilon Western HRP Chemilumiscent substrates, Millipore).

Cell death measurement

Cells were treated either with 1µM Staurosporine (Sigma Aldrich) or with 1µM Actinomycine D (Sigma Aldrich) as indicated, re-suspended in 200µl of buffer (150 mM NaCl, 10 mM HEPES, 5 mM KCl, 1 mM $MgCl_2$, 1.8 mM $CaCl_2$) and incubated with annexin V-FITC (BD Biosciences) for 10 min at RT. A volume of 0.5 µg/ml of DAPI (Molecular Probes) was then added, and samples were analyzed immediately by flow cytometry using a

MACS-Quant Analyzer (Miltenyi Biotec). Anti-Hsp90 antibody (Enzo Life Sciences), anti-PARP, anti-Caspase 3 and anti-Smac antibodies from Cell Signaling Technology were used for western blotting.

DEVDase activity

A total of 20µg of protein (in triplicate) was incubated with 0.2mM of Ac-DEVD-AMC in 50mM HEPES pH 8; 150mM NaCl; 20mM ethylenediaminetetraacetic acid ± 1µM Ac-DEVD-CHO. Caspase activity was determined at 460 nm, and specific activities were expressed in ΔOD per minute and per milligrams of protein.

Mitochondrial membrane potential (ΔΨM) measurement

The transmembrane potential of mitochondria was analyzed with the potential-dependent fluorescent dye TMRE (Molecular Probes/Life Technologies). TMRE was used to label active mitochondria and to determine the mitochondrial membrane potential based on the fluorescence intensity of the TMRE sequestered by the mitochondria. Briefly, patient fibroblast cells were plated in 24-well plates. The cells were treated with 1µM Staurosporine for 24 h. The cells were then incubated with 100nM TMRE for 10 min at 37 °C in the dark. The fluorescence intensity produced by the TMRE was measured by flow cytometry using a MACS-Quant Analyzer (Miltenyi Biotec). Laser excitation of TMRE was at 549 nm and emission was read at 575 nm.

Antibodies Reference

Rabbit anti-CHCHD10 (Sigma, HPA003440), goat anti-CHCHD3 (Abcam, ab99491), mouse anti-CHCHD3 (Abcam, ab69328), goat anti-CHCHD6 [P20] (Santa Cruz Biotechnology, sc-10241), rabbit anti-CHCHD6 (ProSciences, 7133), mouse anti-Mitofilin [2E4AD5] (Abcam, ab110329), rabbit anti-Mitofilin (Abcam, ab48139), rabbit anti-FLAG (Cell Signaling, 2368), mouse anti-DNA [AC-30-10] (Progen, 61014), mouse anti-TFAM (Abnova, H00007019-B01P), mouse anti-Cytochrome *c* (BD Biosciences, 560263), rabbit anti-IgG (NeoMarkers,

Fisher Scientific, NC-100-P0), goat anti-Hsp60 [N20] (Santa Cruz Biotechnology, sc-1052), rabbit anti-Hsp90 (Enzo Life Sciences, ADI-SPA-836), rabbit anti-PARP (Cell Signaling, 9542), rabbit anti-Caspase 3 (Cell Signaling, 9662), rabbit anti-SMAC/DIABLO [D5S3R] (Cell Signaling, 15108), rabbit anti-SMAC/DIABLO (Abcam, ab8114), mouse anti-PCNA (BD Biosciences, 610665), mouse anti-VDAC1 [N152B123] (Millipore, MABN504), rabbit anti-LC3B (Cell Signaling, 2775), mouse anti-NDUFS3 CI (Invitrogen, 439200), mouse anti-NDUFS9 CI (Pierce, PA5-36993), mouse anti-SDHA CII (Invitrogen, 459200), mouse anti-Core 1 CIII (Invitrogen, 459140), mouse anti-COXI CIV (Invitrogen, 459600), MitoProfile® total OXPHOS human WB antibody cocktail (Abcam, ab110411), goat anti-Actin (Santa Cruz Biotechnology, sc-1616), rabbit anti-GAPDH (Abcam, ab9485), goat anti-beta Tubulin (Abcam, ab21057), goat anti-mouse Alexa 488 (Life Technologies, A11099), goat anti-rabbit Alexa 647 (Life Technologies, A21245), goat anti-mouse Immunoglobulins/HRP (DAKO, P0447), rabbit anti-goat Immunoglobulins/HRP (DAKO, P0449), swine anti-rabbit Immunoglobulins/HRP (DAKO, P0399).

LEGENDS TO APPENDIX FIGURES

Appendix Figure S1. Ultrastructural alterations and mitochondrial fragmentation in fibroblasts of patient 2. A. Ultrastructural analysis of control (upper panel) and patient 2 (middle and lower panels) fibroblasts. Scale bar: 1 or 0.5 µm. **B.** Cells obtained from a control and patient 2 were analyzed by confocal microscopy using Mitotracker Red. Scale bar: 10 µm. **C.** Mitochondrial phenotypes showed in B were quantified for 35 randomly-selected individual cells per each studied fibroblast cell line from 2 independent experiments. The data obtained were used to calculate the total length of the mitochondrial network per cell. Differences between the 2 cell lines were analyzed by Student’s t-test (two-sided): extremely significant (***: p=0.0001).

Appendix Figure S2. Fusion analysis in patient fibroblasts. Control and patient fibroblasts expressing mitochondria-targeted photoactivatable GFP (mitoPAGFP) were stained with 7 nM tetramethylrhodamine ethyl ester (TMRE). mitoPAGFP was photoactivated with 405-nm laser in a small region of cells (30 x 30 pixels) at 0 min. Fibroblasts were observed with 15-min intervals for 60 min. Fluorescence intensity of mitoPAGFP was quantified using NIH Image J. Values represent the mean ± S.E.M (n = 7 for control and 9 for patients). Statistical analysis were performed by Student's t-test (two-sided). The results of control and patient 1, that were shown to allow comparison with those of patient 2, were previously published (Bannwarth et al., 2014). Scale bar = 10 µm.

Appendix Figure S3. C*HCHD10* mutant fibroblasts are not defective in mitochondrial fusion. Control and patient cells were infected with lentiviruses expressing wild-type Drp1 or a dominant negative $Drp1^{K38A}$. Cells were subjected to immunofluorescence microscopy using antibodies to Drp1 (red) and the mitochondrial protein Tom20 (green). The expression of $Drp1^{K38A}$ blocked mitochondrial division and elongated mitochondrial tubules in the control and patient cells. Nuclei were visualized by DAPI staining (blue). Boxed regions show magnified images. Scale bar: 20 µm.

Appendix Figure S4. Decrease of nucleoid number in patient fibroblasts shown by using anti-TFAM antibodies. A. In control and patient fibroblasts, nucleoids were labeled with antibodies against TFAM and mitochondria were stained with Mitotracker. Image analysis was performed by confocal microscopy. Scale bar: 10 µm. **B-C.** The average number (B) and size (C) of nucleoids, labeled with antibodies against TFAM shown in A, were quantified for 35 randomly-selected individual cells per each studied fibroblast cell line from 2 independent experiments. Differences between the cell lines were analyzed by Student's t-test (two-sided): extremely significant (***: p=0.0001).

Appendix Figure S5. Expression level of OXPHOS proteins, encoded either by mtDNA or by nuclear genes, is not affected in patient fibroblasts. Representative western blot of ATP5A, UQCRC2, COX I, SDHB, COX II and NDUFB8 proteins performed with fibroblast lysates obtained from control (C) and patients (P1, P2). Hsp60 and GAPDH are also shown as controls. ns, non specific; * indicates proteins encoded by mtDNA.

Appendix Figure S6. Overexpression of the *CHCHD10^{P34S}* allele in HeLa cells leads to fragmentation of the mitochondrial network. Transfections were performed with empty vector (EV) or vectors encoding either wild-type CHCHD10-FLAG (WT) or mutant CHCHD10-FLAG (P34S). **A.** Western blot on HeLa cells extracts using antibodies against FLAG or GAPDH. **B.** Analysis of Mitotracker (red) staining and CHCHD10-FLAG (green) immuno-labeling by fluorescence microscopy in HeLa cells transfected with empty vector (EV), wild-type CHCHD10-FLAG (WT) or mutant CHCHD10-FLAG (P34S). Scale bar: 10 µm. **C.** Quantification of mitochondrial phenotypes of cells transfected with empty vector (EV) or vectors encoding either wild-type CHCHD10-FLAG (WT) or mutant CHCHD10-FLAG (P34S). Thirty five randomly-selected individual cells per each transfection were analyzed from 2 independent experiments. The data obtained were used to calculate the total length of the mitochondrial network per cell. Differences between the cells tranfected were analyzed by Student's t-test (two-sided): extremely significant (***: p=0.0001).

Appendix Figure S7. Overexpression of the *CHCHD10^{P34S}* allele in HeLa cells leads to defect in cristae maintenance. A. Representative image of mitochondria in cells overexpressing the *CHCHD10^{WT}* allele. **B-C.** Representative images of mitochondria in cells overexpressing the mutant *CHCHD10 ^{P34S}* allele. Scale bar = 1 µm.

Appendix Figure S8. Decrease of nucleoid number in HeLa cells overexpressing the *CHCHD10^{S59L}* and *CHCHD10^{P34S}* alleles shown by using anti-TFAM antibodies. A. Transfections were performed with empty vector (EV) or vectors encoding wild-type

CHCHD10-FLAG (WT), CHCHD10-FLAG (S59L) or CHCHD10-FLAG (P34S) mutants. Mitochondria were stained with Mitotracker. Cells overexpressing wild-type and mutant CHCHD10 were labeled with anti-FLAG antibody. Nucleoids were visualized with an antiserum against TFAM. Image analysis was performed by confocal microscopy. Scale bar: 10 µm. **B-C.** The average number (B) and size (C) of nucleoids, labeled with antibody against TFAM shown in A, were quantified for 35 randomly-selected individual cells per each studied cell line from 2 independent experiments. Differences between the cell lines were analyzed by Student's t-test (two-sided): very significant (**: $0.01>p>0.001$) or extremely significant (***: $p<0.001$). Nucleoid number: S59L versus WT: ***: $p=0.0001$, P34S versus WT: ***: $p=0.0010$. Nucleoid surface: P34S versus WT: **: $p=0.0028$.

Appendix Figure S9. Delayed cytochrome *c* release in C*HCHD10* mutant fibroblasts compared to control fibroblasts. Control and patient (P1, P2) fibroblasts were treated with 1µM of staurosporine (STS) for 6 h. Treated and untreated cells were immuno-labelled with anti-cytochrome *c* (upper panels) and stained with Mitotracker (medium panels). Overlays of fluorescence are shown in lower panels. Scale bar: 10 µm.

Appendix Figure S10. Delayed cytochrome *c* release in HeLa cells expressing C*HCHD10* mutant alleles. Transfections were performed with vectors encoding either wild-type CHCHD10-FLAG (WT) or mutant CHCHD10-FLAG (P34S and S59L) and cells were treated with 1 µM Actinomycin D (ActD) for 3 h. Treated and untreated cells were immuno-labelled with anti-cytochrome *c* and stained with Mitotracker. Overlays of fluorescence are shown. Transfected cells were revealed by immuno-labelling with anti-FLAG antibody. Scale bar: 10 µm.

Appendix Table S1. Respiratory chain analysis in fibroblasts of patient 2. Spectrophotometric analysis of the respiratory chain enzyme activities in patient fibroblasts in glucose (A) and in galactose medium (B). CS, citrate synthase. Results are expressed as

extreme absolute values or absolute values for controls or patients, respectively. Values are expressed in nanomols of substrate per minute per milligram of proteins (lowered values are in grey). **C-D.** Polarographic analysis of the respiratory chain in patient fibroblasts in glucose (C) and in galactose medium (D). G3P, glycerol 3-phosphate. Results are expressed as extreme absolute values or absolute values for controls or patients, respectively. Values are expressed in nanomols of oxygen per minute per milligram of proteins (lowered values are in grey).

A

SPECTROPHOTOMETRIC ANALYSIS ON FIBROBLASTS						
**GLUCOSE MEDIUM**						
**Enzymatic activities**	**I**	**II**	**III**	**IV**	**V**	**CS**
Control values (nmol/min/mg of proteins)	9.0-27.1	18.5-54.0	57.4-176.2	109.9-350.0	22.0-46.2	74.7-161.1
Patient 2	13.8	22.2	119.0	143.5	38.6	114.7

B

SPECTROPHOTOMETRIC ANALYSIS ON FIBROBLASTS						
**GALACTOSE MEDIUM**						
**Enzymatic activities**	**I**	**II**	**III**	**IV**	**V**	**CS**
Control values (nmol/min/mg of proteins)	15.2-19.4	28.2-33.1	88.8-116.4	181.7-315.4	22.7-32.1	124.8-223.0
Patient 2	**9.4**	**20.6**	**85.6**	**146.8**	32.9	198.5

C

OXYGRAPHIC ANALYSIS ON FIBROBLASTS				
**GLUCOSE MEDIUM**				
**Oxygen consumption**	**Intact cells**	**Digitonin permeabilized cells**		
		**Glutamate+Malate**	**Succinate**	**G3P**
Control values (nmol O2/min/mg of proteins)	5.90 - 13.80	8.00 - 16.60	8.00 - 15.80	4.90 - 13.50
Patient 2	10.58	10.25	12.82	10.03

D

OXYGRAPHIC ANALYSIS ON FIBROBLASTS				
**GALACTOSE MEDIUM**				
**Oxygen consumption**	**Intact cells**	**Digitonin permeabilized cells**		
		**Glutamate+Malate**	**Succinate**	**G3P**
Control values (nmol O2/min/mg of proteins)	5.58 – 8.25	8.16 – 9.91	8.60 – 9.76	5.22 – 12.91
Patient 2	7.98	9.26	9.24	8.08

Appendix Table S1

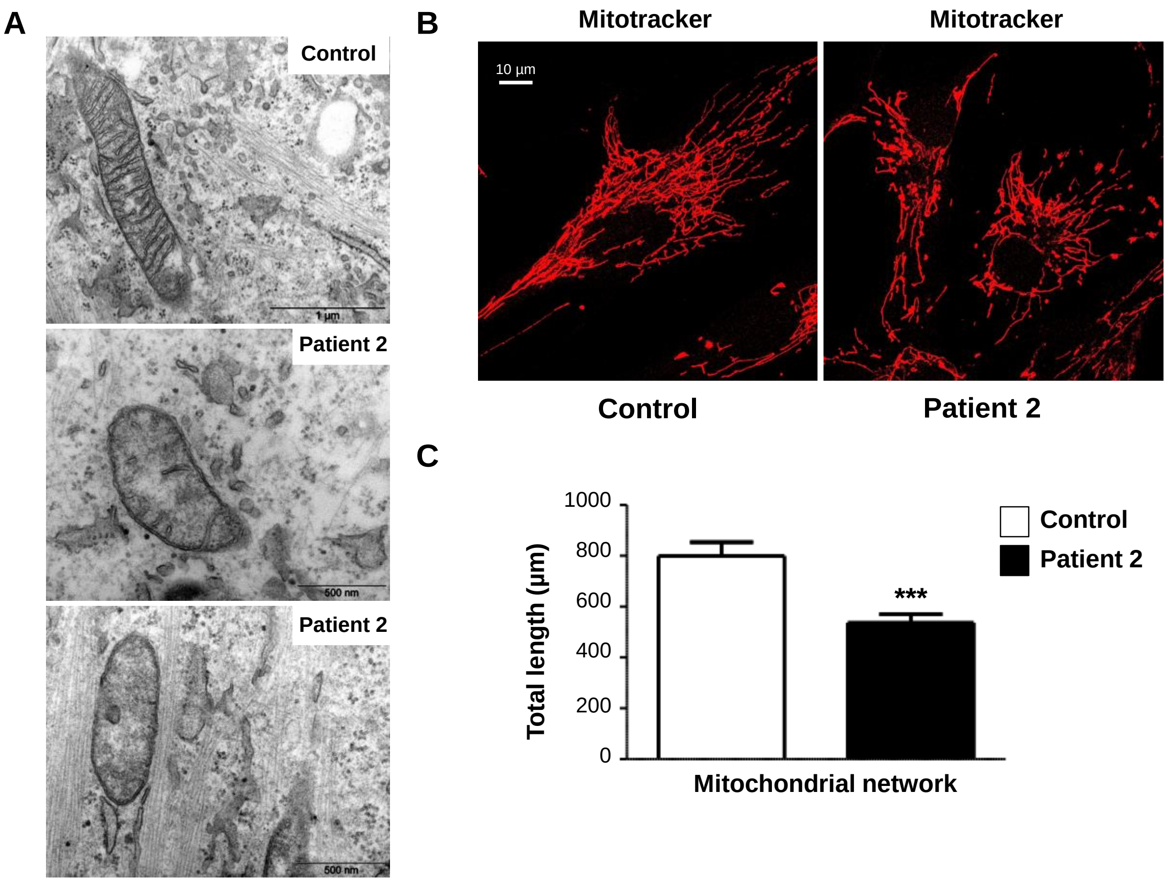

Appendix Figure S1

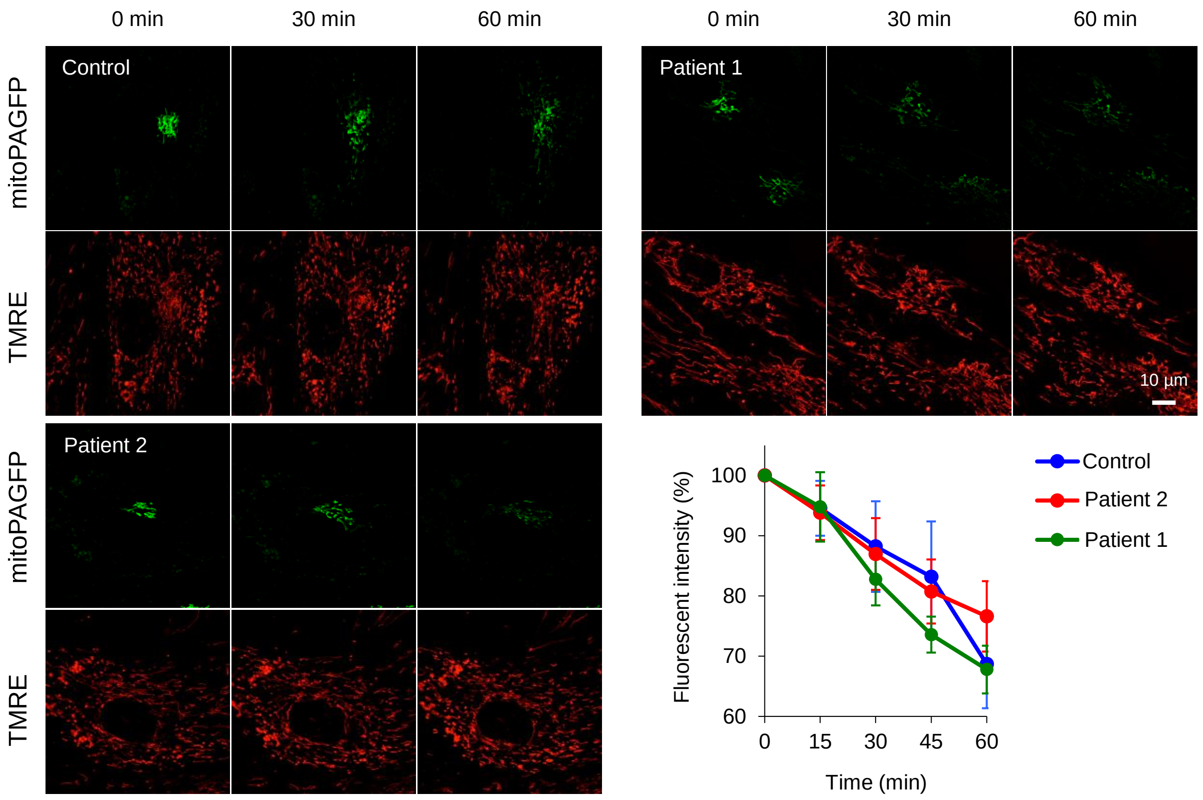

Appendix Figure S2

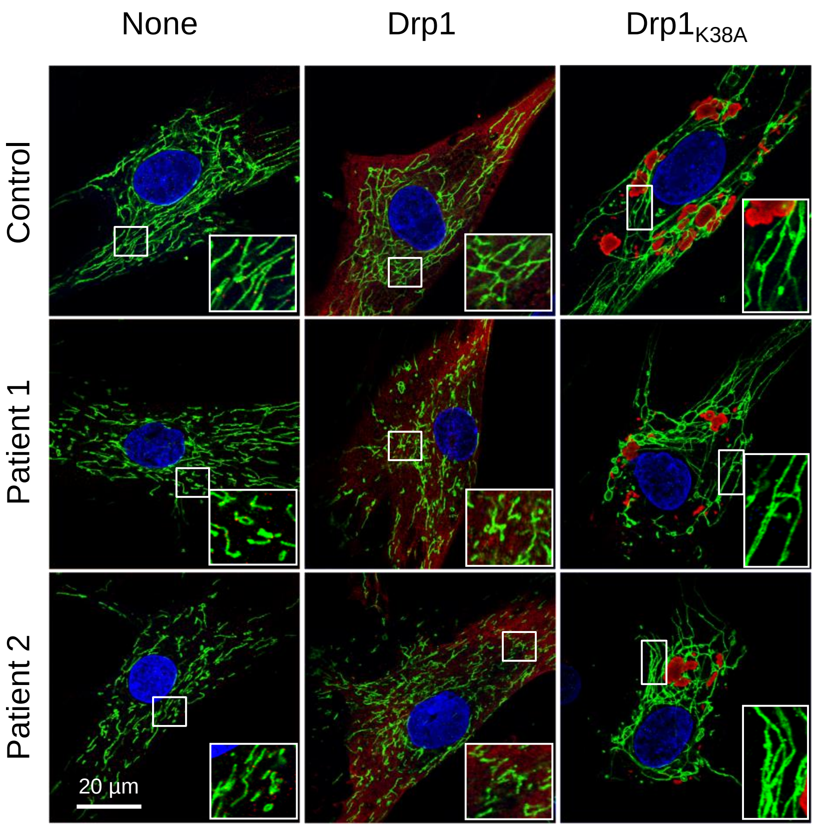

Appendix Figure S3

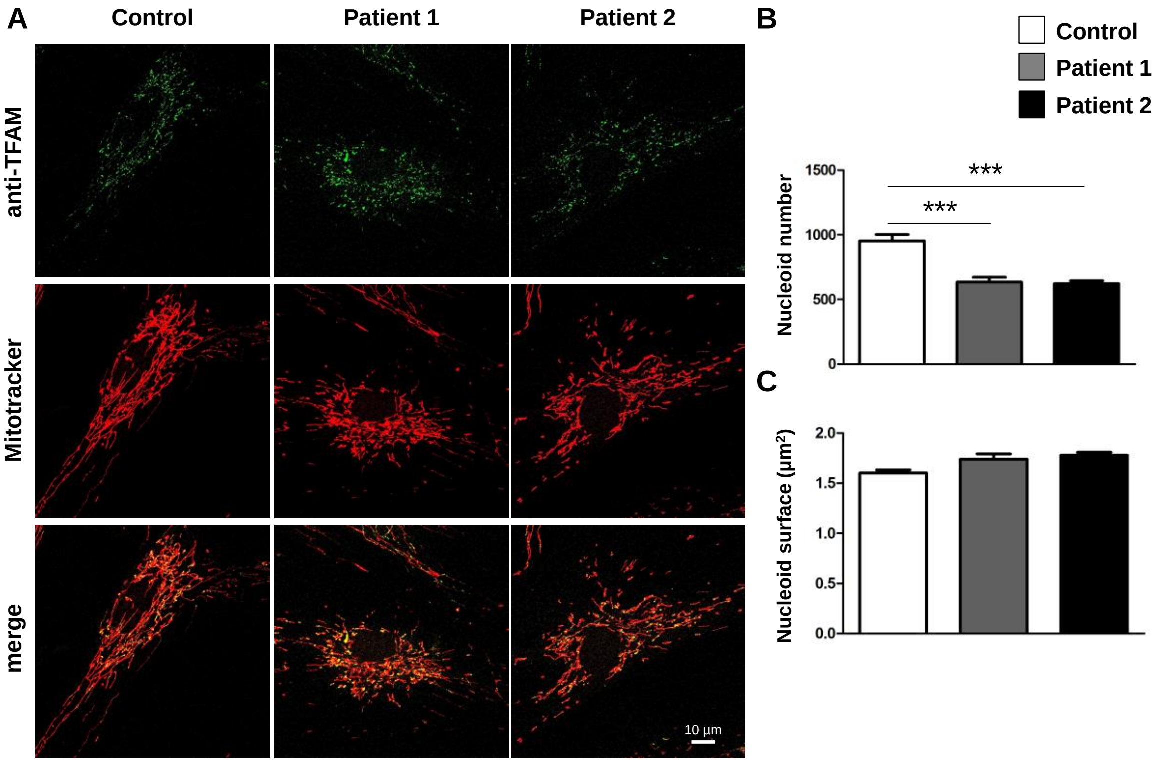

Appendix Figure S4

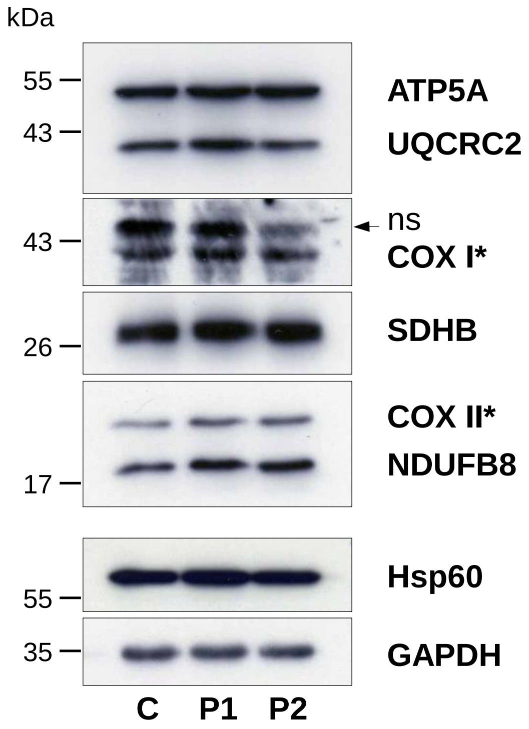

Appendix Figure S5

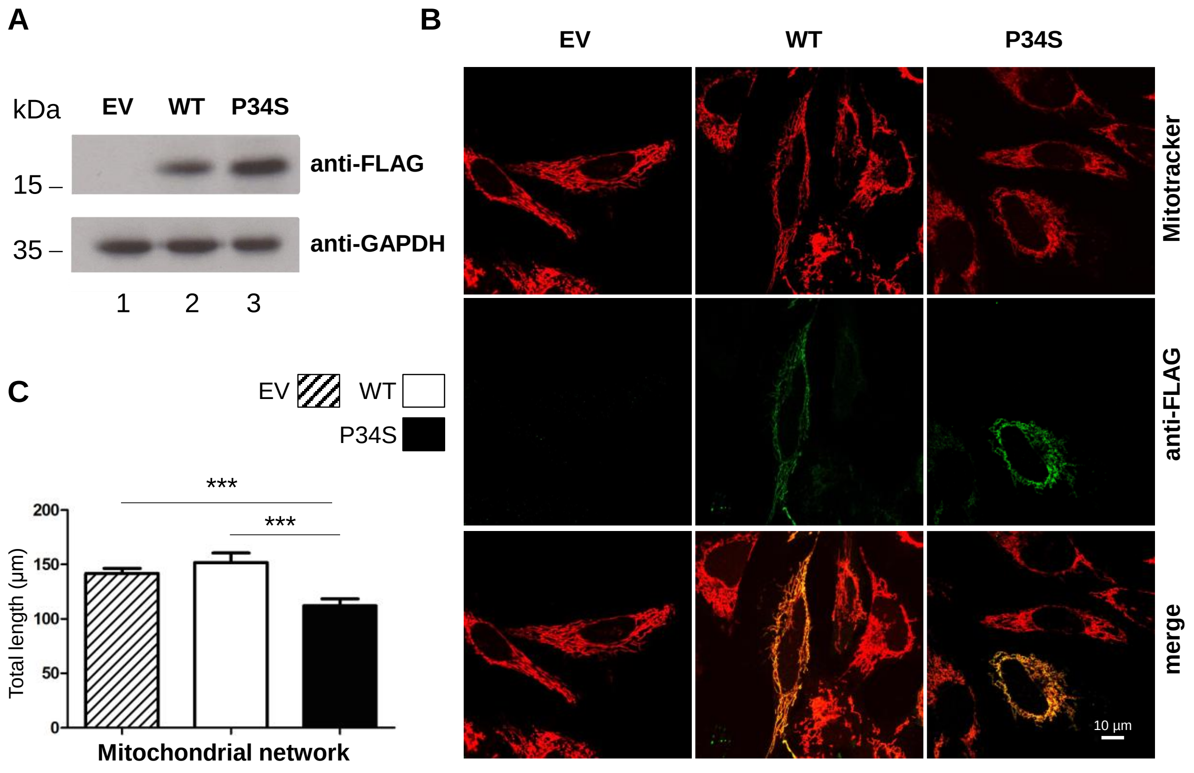

Appendix Figure S6

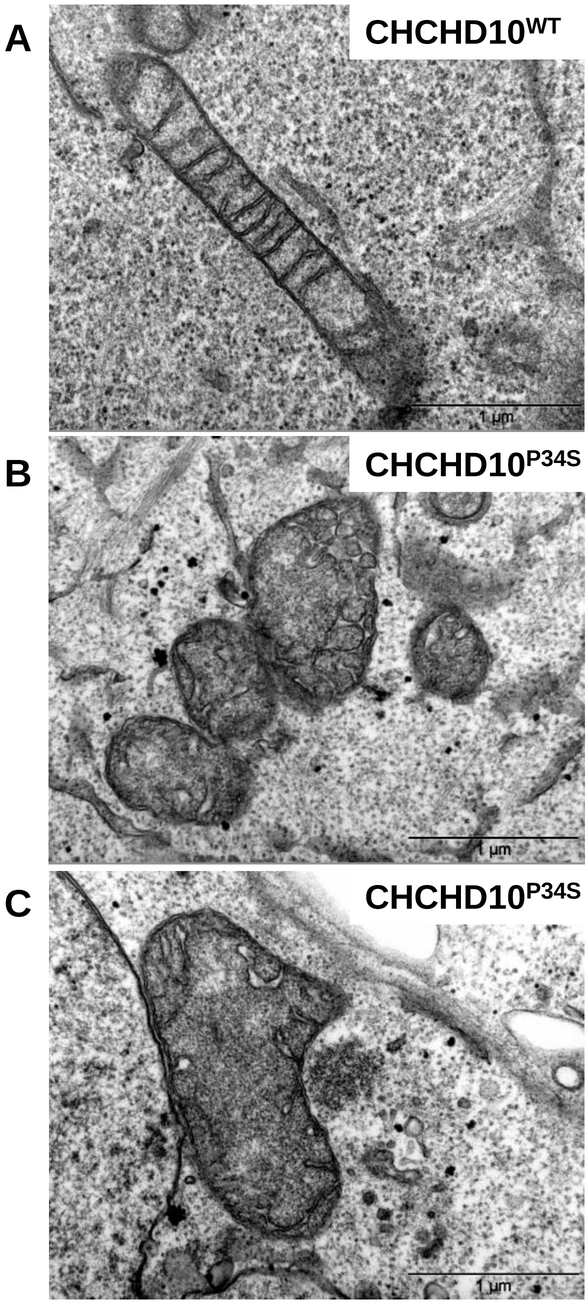

Appendix Figure S7

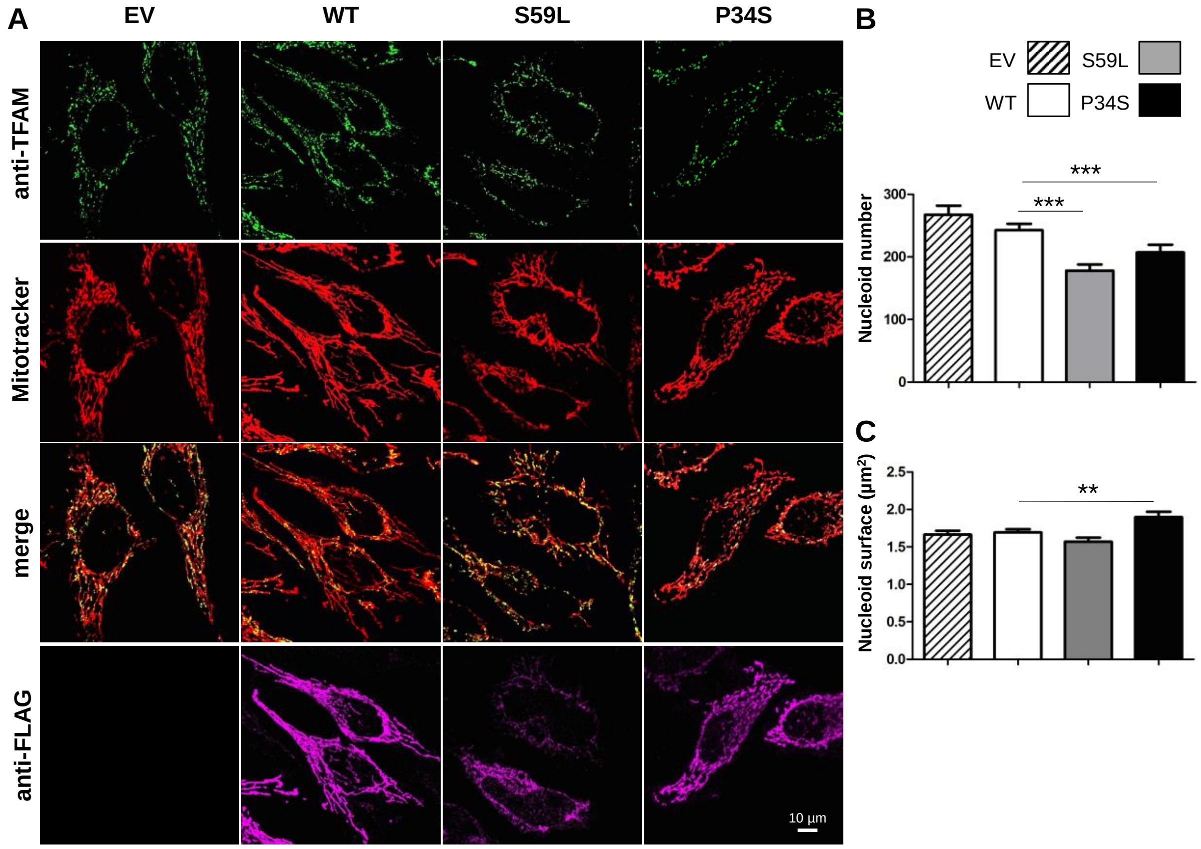

Appendix Figure S8

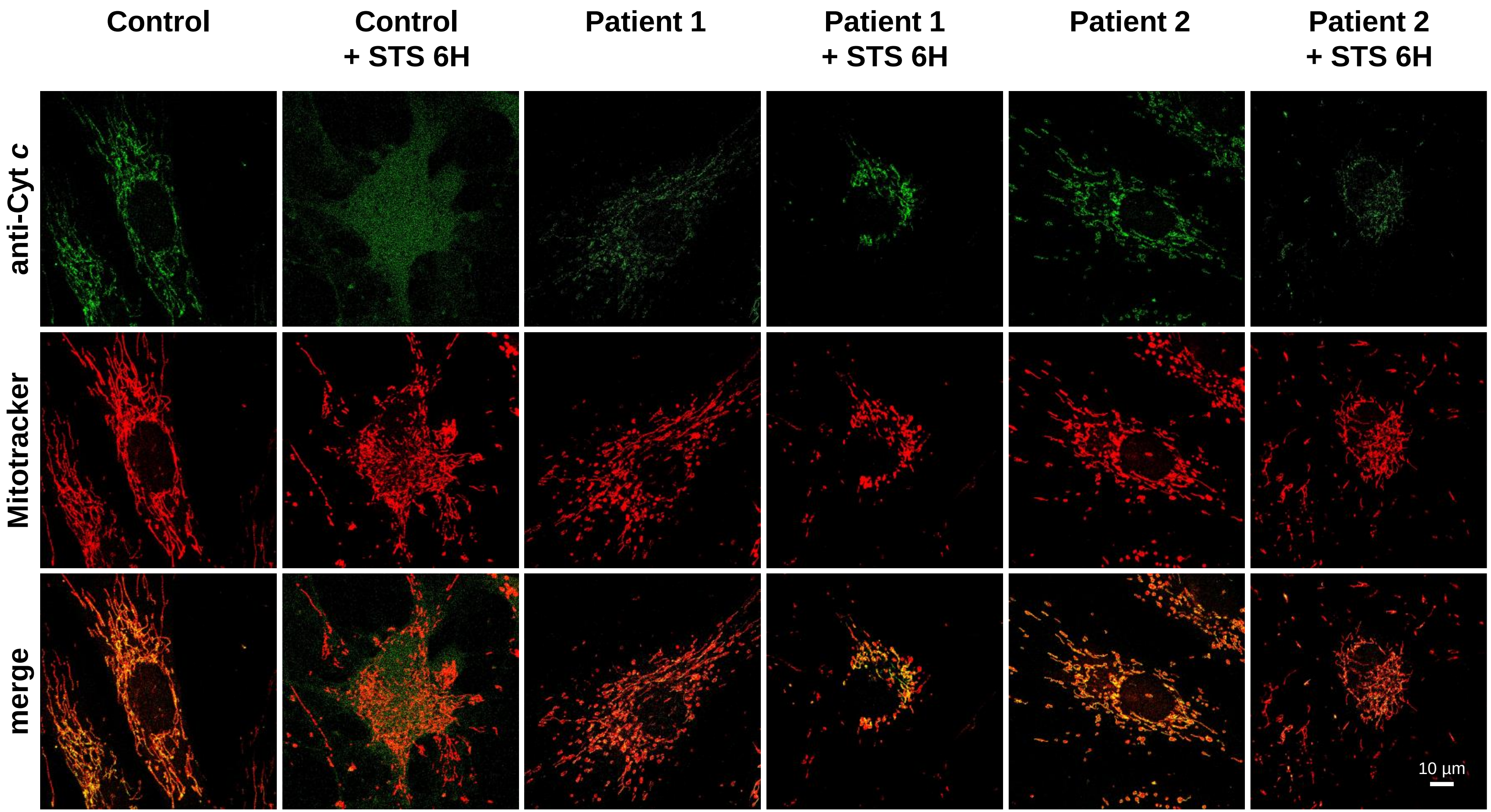

Appendix Figure S9

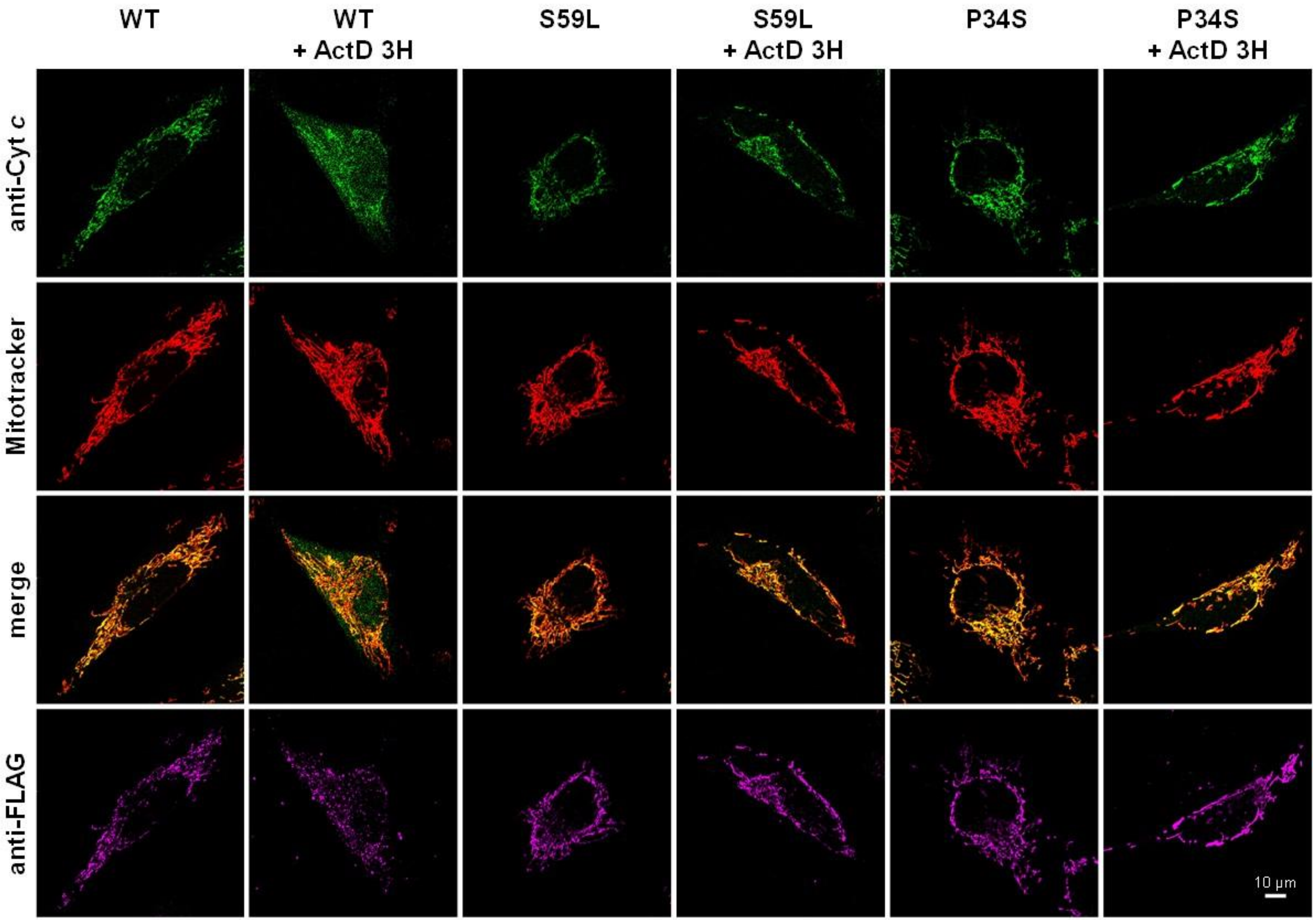

Appendix Figure S10

APPENDIX REFERENCES

Ballinger, S.W., Van Houten, B., Jin, G.F., Conklin, C.A. and Godley, B.F. (1999) Hydrogen peroxide causes significant mtDNA damage in human RPE cells. Exp. Eyes Res. 68: 765-772.

Bannwarth, S., Ait-El-Mkadem, S., Chaussenot, A., Genin, E.C., Lacas-Gervais, S., Fragaki, K., Berg-Alonso, L., Kageyama, Y., Serre, V., Moore, D.G., Verschueren, A., Rouzier, C., Le Ber, I., Augé, G., Cochaud, C., Lespinasse, F., N'Guyen, K., de Septenville, A., Brice, A., Yu-Wai-Man, P., Sesaki, H., Pouget, J. and Paquis-Flucklinger, V. (2014) A mitochondrial origin for frontotemporal dementia and amyotrophic lateral sclerosis through CHCHD10 involvement. Brain 137: 2329-2345.

Bannwarth, S., Figueroa, A., Fragaki, K., Destroismaisons, L., Lacas-Gervais, S., Lespinasse, F., Vandenbos, F., Pradelli, L.A., Ricci, J.E., Rötig, A., Michiels, J.F., Vande Velde, C. and Paquis-Flucklinger, V. (2012) The human MSH5 (MutSHomolog 5) protein localizes to mitochondria and protects the mitochondrial genome from oxidative damage. Mitochondrion 12: 654-665.

Hunter, S.E., Jung, D., Di Giulio, R.T. and Meyer, J.N. (2010) The QPCR assay for analysis of mitochondrial DNA damage, repair, and relative copy number. Methods. 51: 444-451.

Karbowski, M., Arnoult, D., Chen, H., Chan, D.C., Smith, C.L. and Youle, R.J. (2004) Quantitation of mitochondrial dynamics by photolabeling of individual organelles shows that mitochondrial fusion is blocked during the Bax activation phase of apoptosis. J. Cell. Biol. 164: 493-499.

Kim, J.S., Xu, X., Li, H., Solomon, D., Lane, W.S., Jin, T. and Waldman, T. (2011) Mechanistic analysis of a DNA damage-induced, PTEN-dependent size checkpoint in human cells. Mol. Cell. Biol. 31: 2756-2771.

Rustin, P., Chrétien, D., Bourgeron, T., Gérard, B., Rotig, A., Saudubray, J.M. and Munnich, A. (1994) Biochemical and molecular investigations in respiratory chain deficiency. Clin. Chim. Acta. 228: 35-51.

Santos, J., Meyer, J., Mandavilli, B. and Van Houten, B. (2006) Quantitative PCR-based measurement of nuclear and mitochondrial DNA damage and repair in mammalian cells. Methods Mol Biol. 314: 183-199.

Shull, S., Heintz, N.H., Periasamy, M., Manohar, M., Janssen, Y.M., Marsh, J.P. and Mossman, B.T. (1991) Differential regulation of antioxidant enzymes in response to oxidants. J Biol Chem 266: 24398-24403.

Wakabayashi, J., Zhang, Z., Wakabayashi, N., Tamura, Y., Fukaya, M., Kensler, T.W., Iijima, M. and Sesaki, H. (2009) The dynamin-related GTPase Drp1 is required for embryonic and brain development in mice. J. Cell. Biol. 186: 805-816.

Yakes, F.M. and Van Houten, B. (1997) Mitochondrial DNA damage is more extensive and persists longer than nuclear DNA damage in human cells following oxidative stress. Proc. Natl. Acad. Sci. USA 94: 514-519.

Zanna, C., Ghelli, A., Porcelli, A.M., Karbowski, M., Youle, R.J., Schimpf, S., Wissinger, B., Pinti, M., Cossarizza, A., Vidoni, S., Valentino, M.L., Rugolo, M. and Carelli, V. (2008) OPA1 mutations associated with dominant optic atrophy impair oxidative phosphorylation and mitochondrial fusion. Brain. 131: 352-367.
