# Supplementary figures and images for: CHCHD10 mutations promote loss of mitochondrial cristae junctions with impaired mitochondrial genome maintenance and inhibition of apoptosis
Source: EMBO Mol Med. 2015 Dec 14;8(1):58–72. doi: 10.15252/emmm.201505496 (PMC4718158; doi:10.15252/emmm.201505496)

**Figure 1 C**

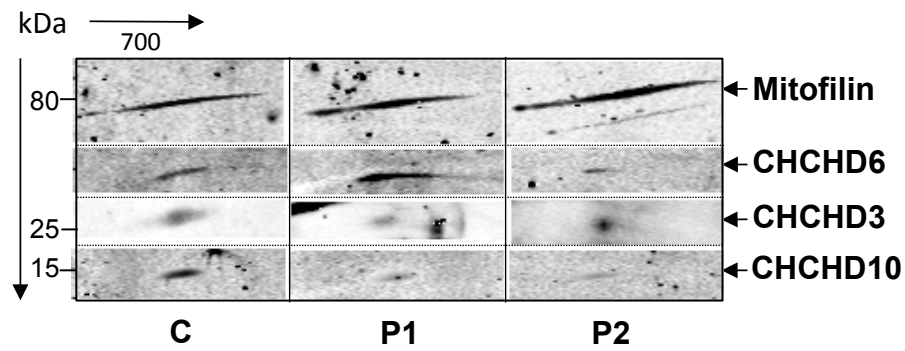

**Uncropped scans Figure 1 C**

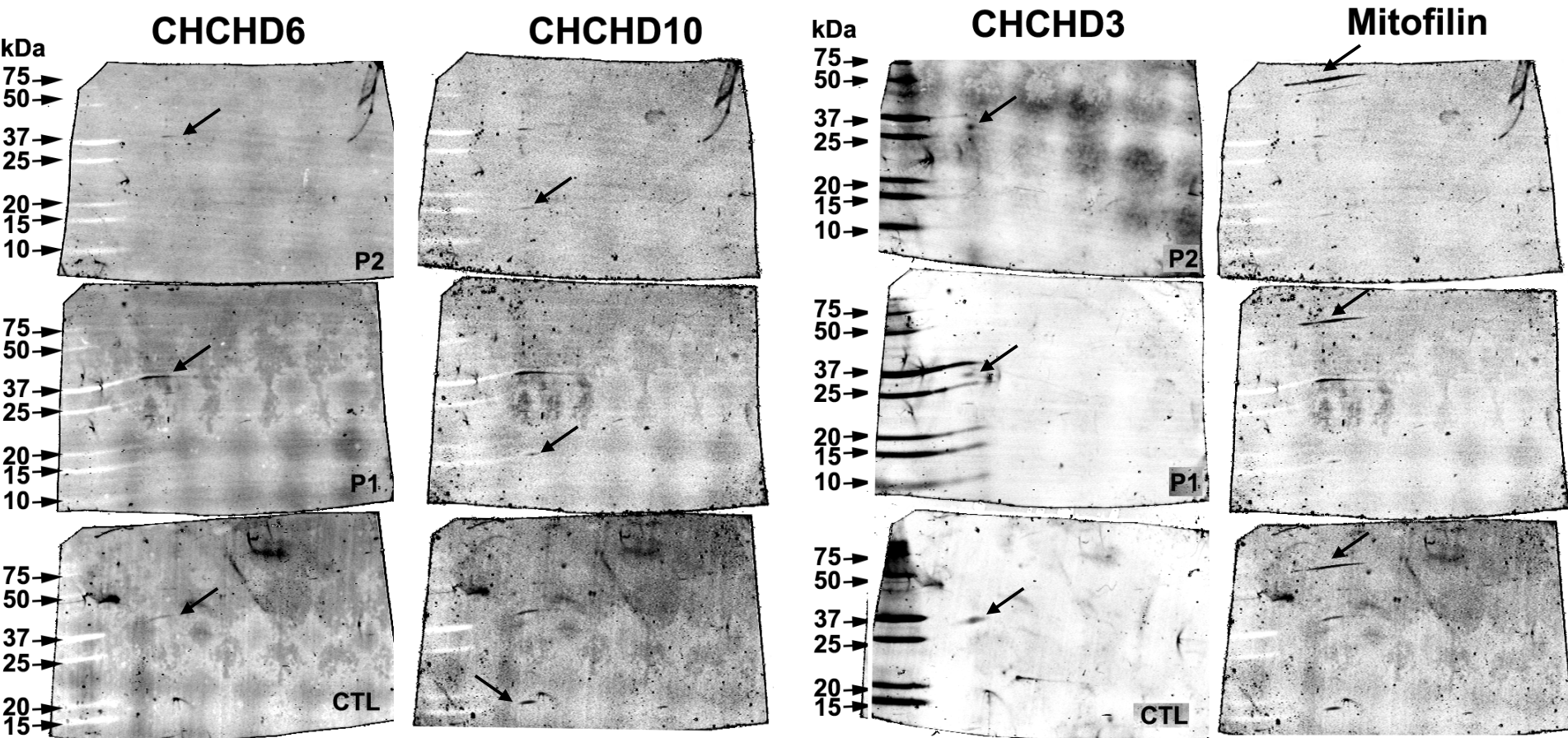

Supplement: Supplementary file 3 — Source Data for Figure 1 [file EMMM-8-58-s002.pdf]

Figure 2 A

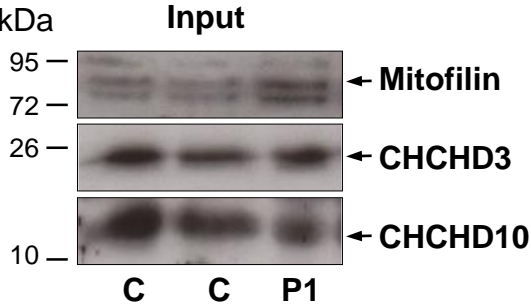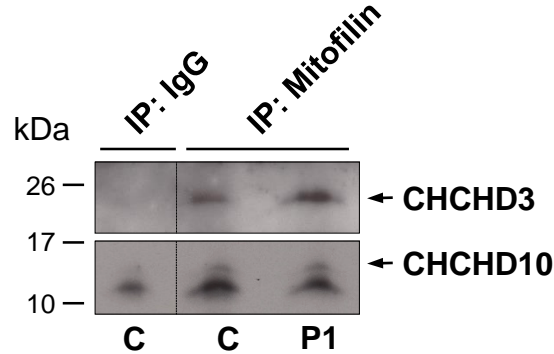

Uncropped scans Figure 2 A

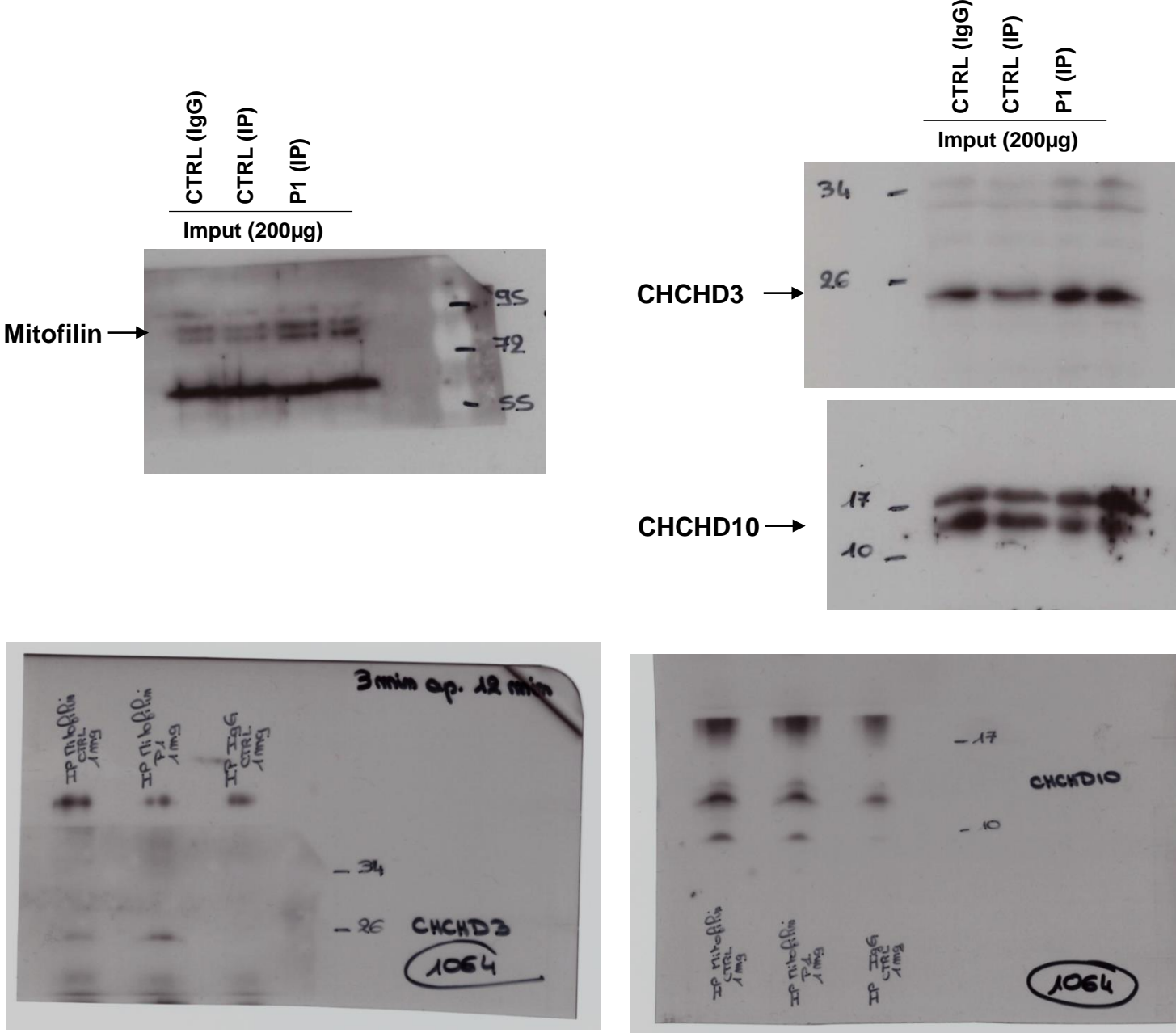

Figure 2 A

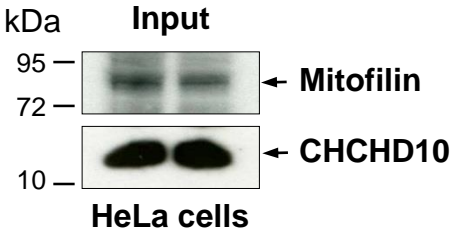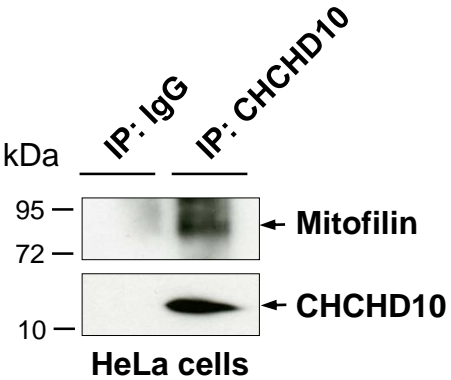

Uncropped scans Figure 2 A

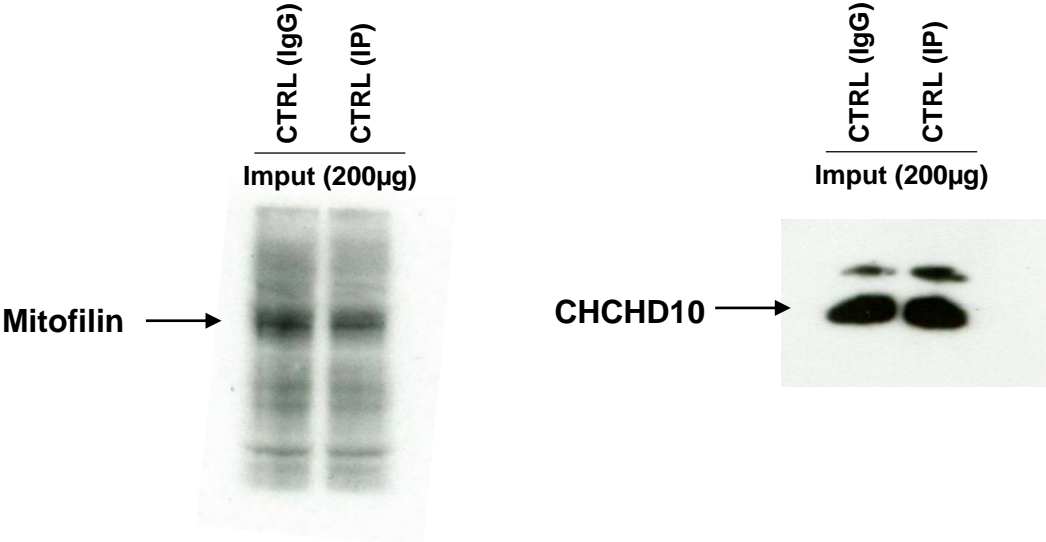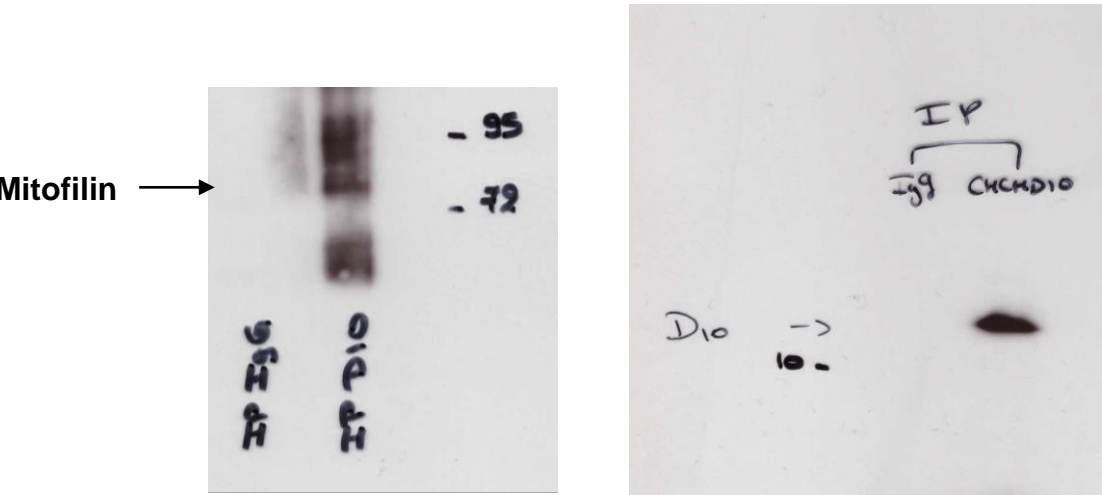

Supplement: Supplementary file 4 — Source Data for Figure 2 [file EMMM-8-58-s003.pdf]

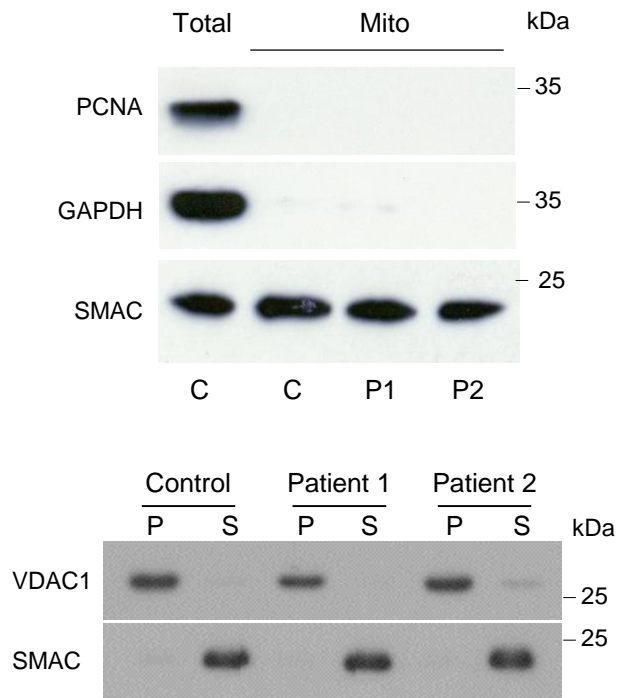

**Figure 5**

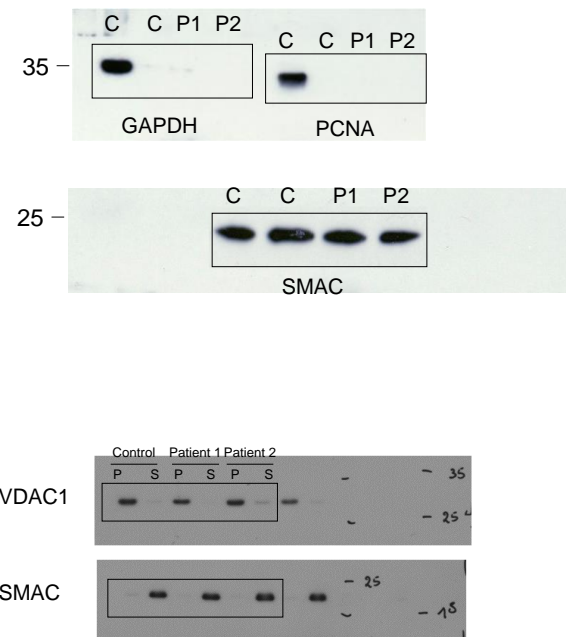

**Uncropped scans  
Figure 5**

Supplement: Supplementary file 5 — Source Data for Figure 5 [file EMMM-8-58-s004.pdf]

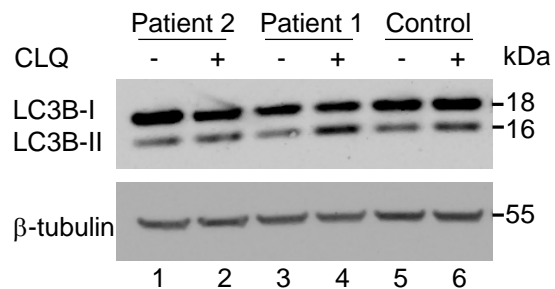

**Figure 6**

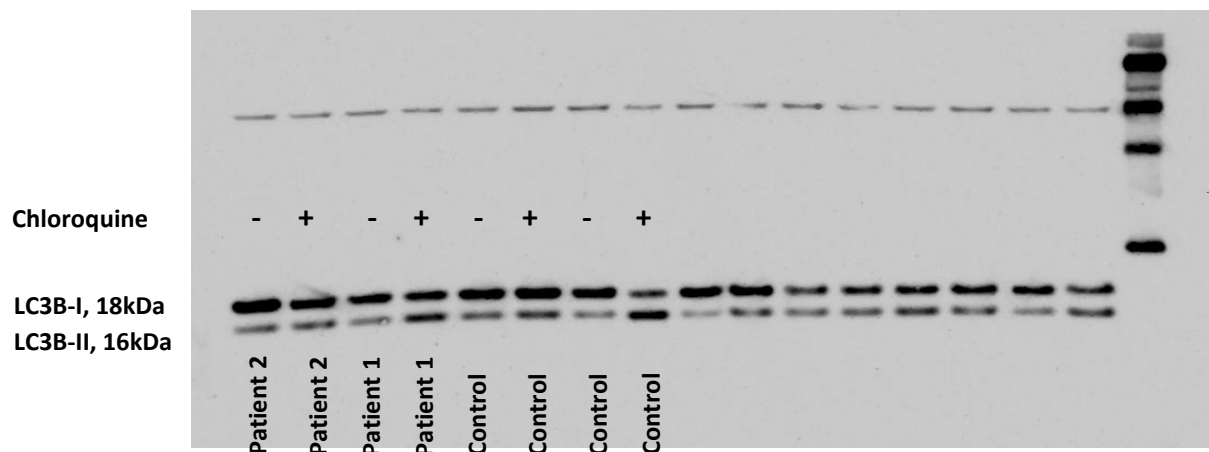

**Uncropped scans  
Figure 6**

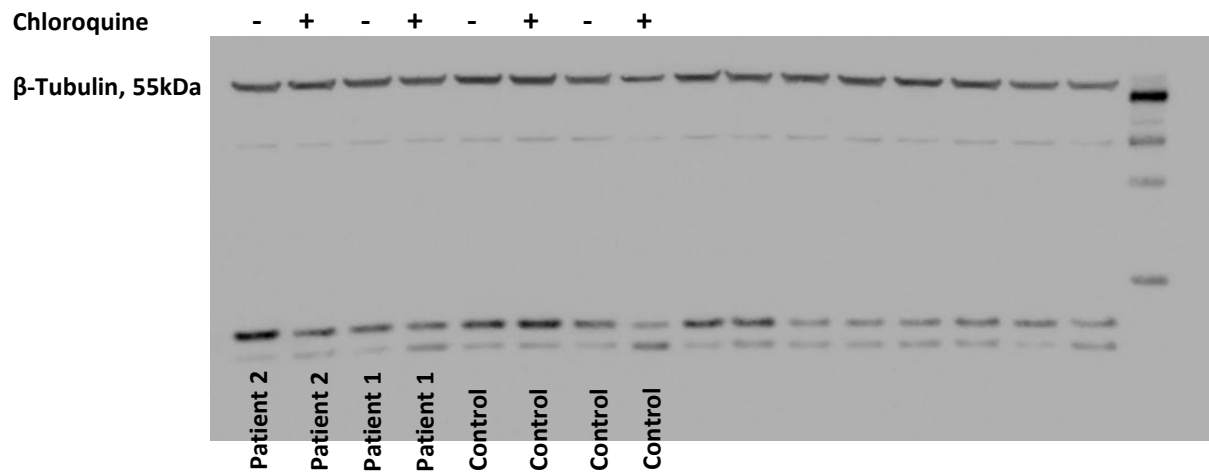

Supplement: Supplementary file 6 — Source Data for Figure 6 [file EMMM-8-58-s005.pdf]

Figure 7

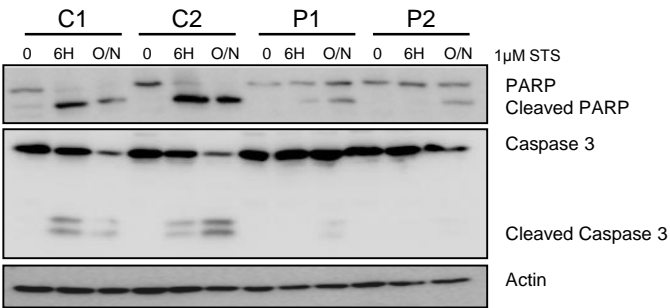

Uncropped scans  
Figure 7

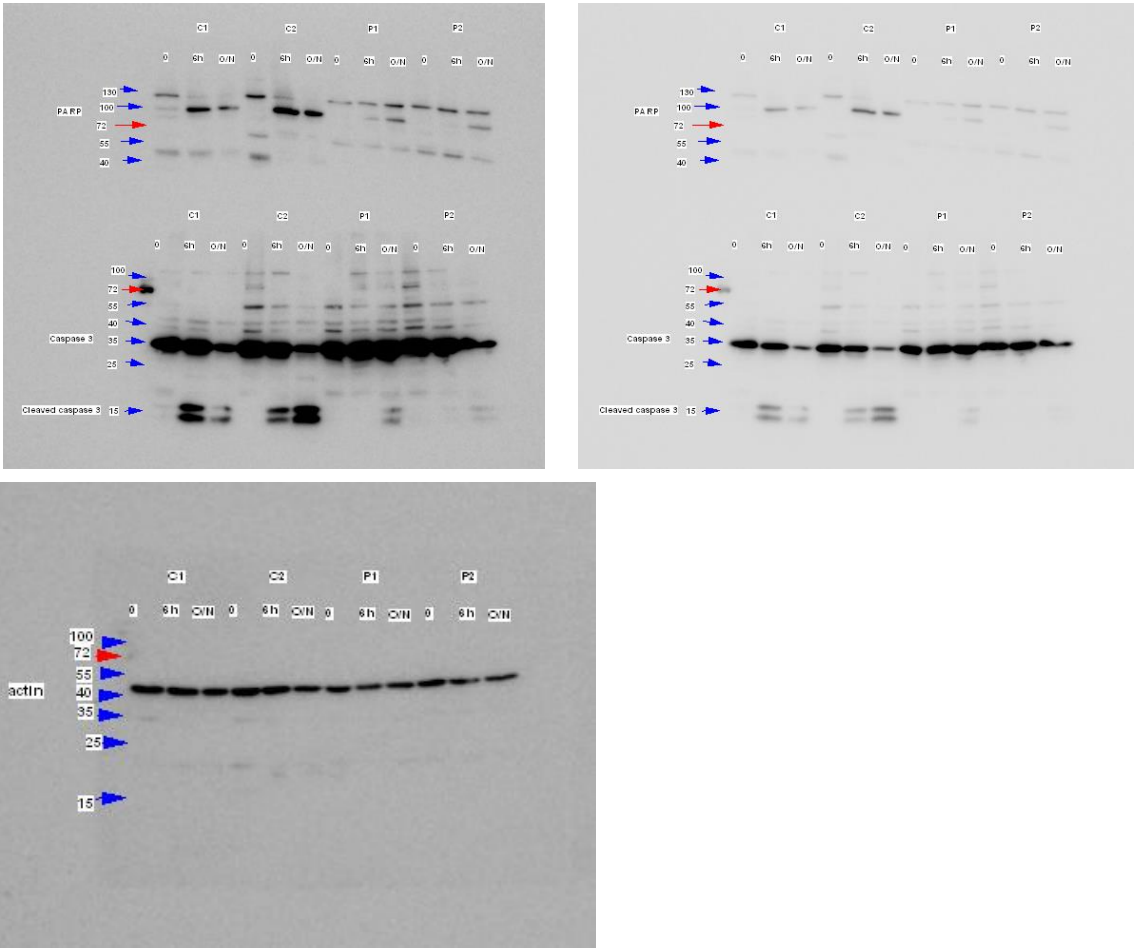

Supplement: Supplementary file 7 — Source Data for Figure 7 [file EMMM-8-58-s006.pdf]

Figure 8 A

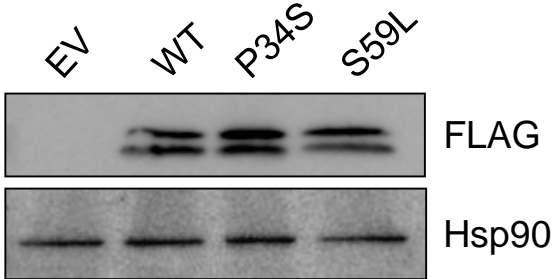

Uncropped scans  
Figure 8 A

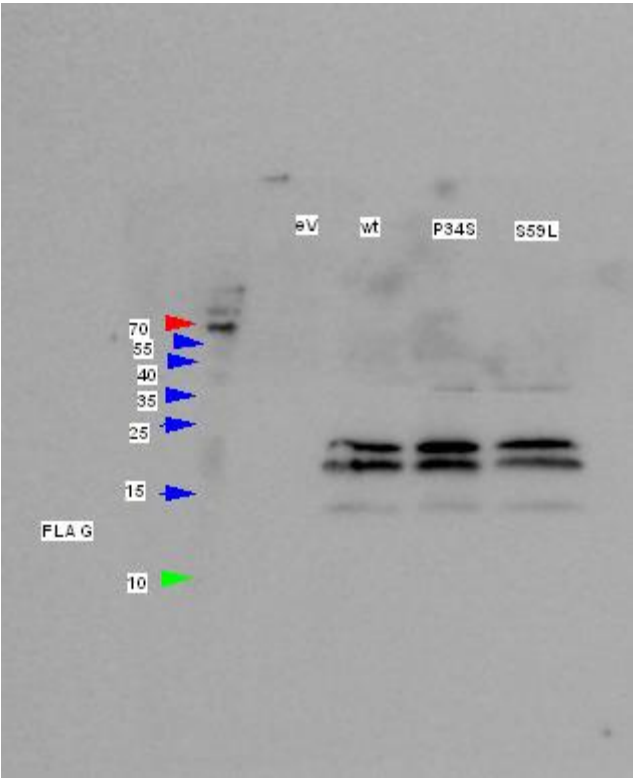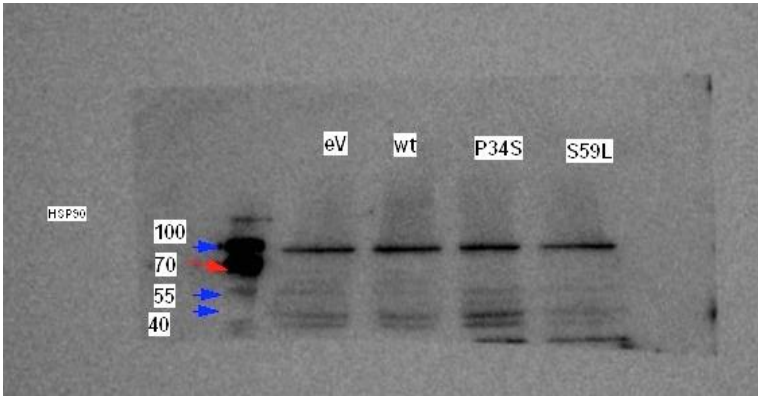

Figure 8 D

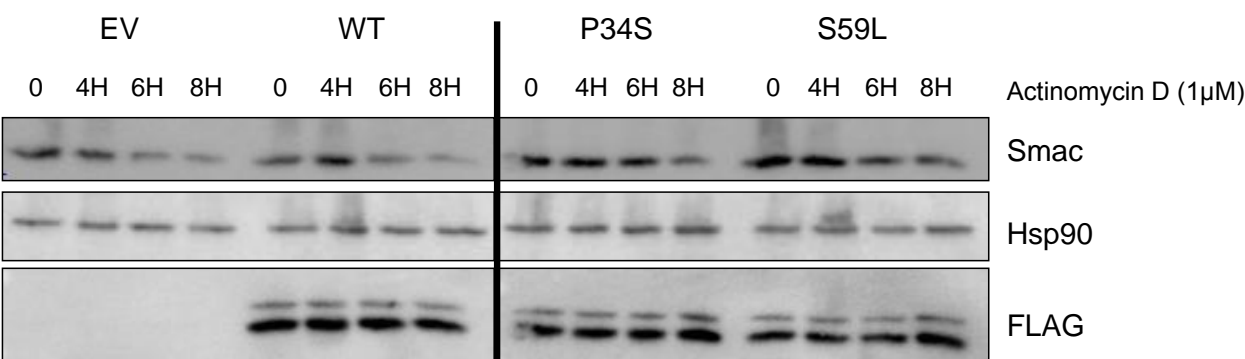

Uncropped scans  
Figure 8 D

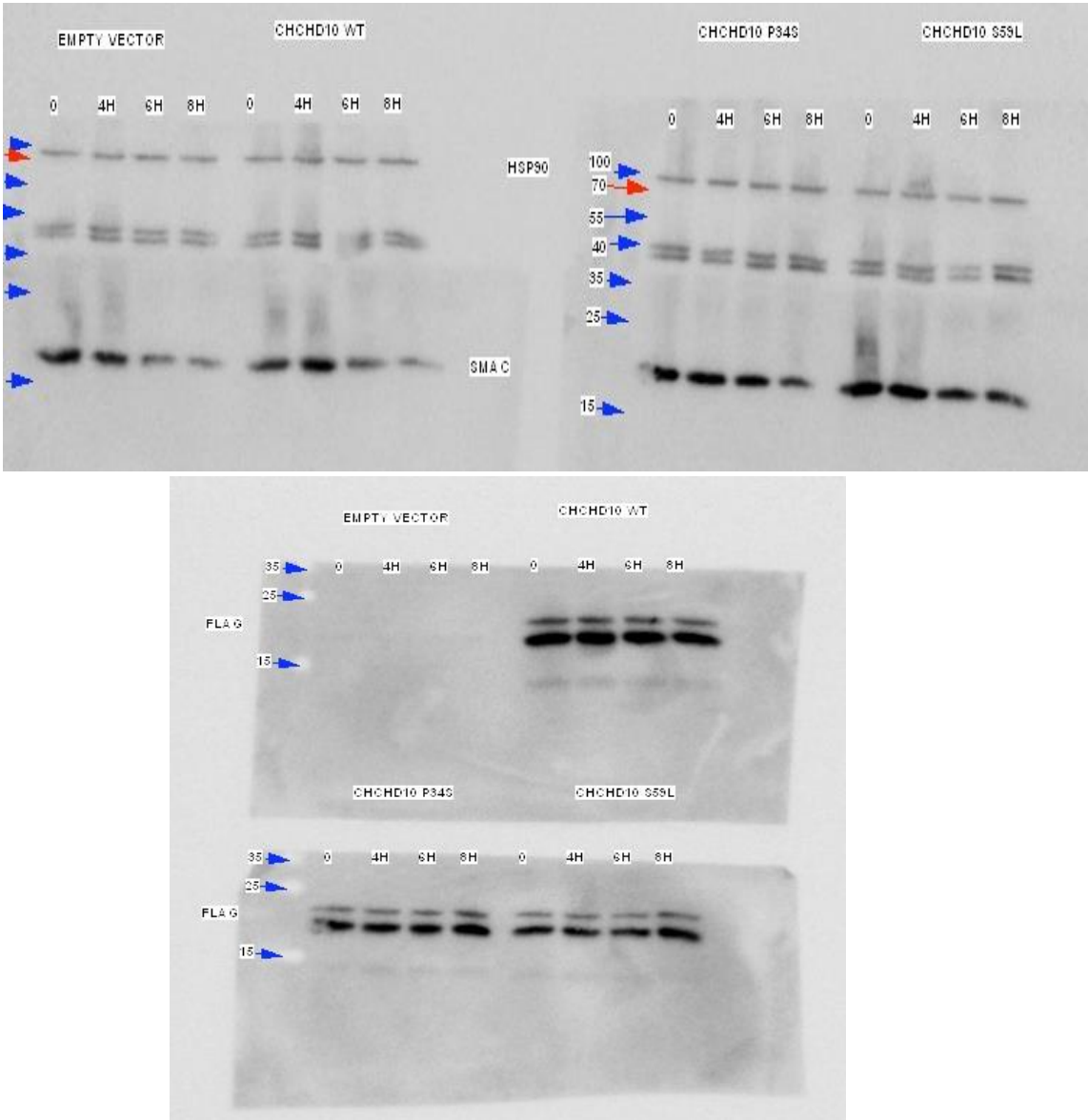

Supplement: Supplementary file 8 — Source Data for Figure 8 [file EMMM-8-58-s007.pdf]
